# 3D nanorhombus nickel nitride as stable and cost-effective counter electrodes for dye-sensitized solar cells and supercapacitor applications

**DOI:** 10.1039/c8ra00347e

**Published:** 2018-02-27

**Authors:** Saradh Prasad, G. Durai, D. Devaraj, Mohamad Saleh AlSalhi, J. Theerthagiri, Prabhakarn Arunachalam, M. Gurulakshmi, M. Raghavender, P. Kuppusami

**Affiliations:** Department of Electrical and Electronics Engineering, School of Electronics and Electrical Technology (SEET), Kalasalingam Academy of Research and Education (KARE) Krishnankoil, Virudhunagar 626126 Tamil Nadu India deva230@yahoo.com; Research Chair on Laser Diagnosis of Cancers, Department of Physics and Astronomy, College of Science, King Saud University 11451 Riyadh Saudi Arabia malsalhy@gmail.com; Centre of Excellence for Energy Research, Sathyabama Institute of Science and Technology (Deemed to be University) Chennai 600119 India; Electrochemistry Research Group, Chemistry Department, College of Science, King Saud University Riyadh 11451 Saudi Arabia; Department of Physics, Yogi Vemana University Kadapa-516003 A.P. India

## Abstract

Transition metal nitride based materials have attracted significant interest owing to their excellent properties and multiple applications in the field of electrochemical energy conversion and storage devices. Herein we synthesize 3D nanorhombus nickel nitride (Ni_3_N) thin films by adopting a reactive radio frequency magnetron sputtering process. The as-deposited 3D nano rhombus Ni_3_N thin films were utilized as cost-effective electrodes in the fabrication of supercapacitors (SCs) and dye-sensitized solar cells (DSSCs). The structure, phase formation, surface morphology and elemental composition of the as-deposited Ni_3_N thin films were characterized by X-ray diffraction (XRD), field emission scanning electron microscopy (FESEM), energy-dispersive X-ray spectroscopy (EDS) and atomic force microscopy (AFM). The electrochemical supercapacitive performance of the Ni_3_N thin films was examined by cyclic voltammetry (CV) and galvanostatic charge–discharge (GCD) techniques, in 3 M KOH supporting electrolyte. The areal capacitance of the Ni_3_N thin film electrode obtained from CV analysis was 319.5 mF cm^−2^ at a lower scan rate of 10 mV s^−1^. Meanwhile, the Ni_3_N thin film showed an excellent cyclic stability and retained 93.7% efficiency of its initial capacitance after 2000 cycles at 100 mV s^−1^. Interestingly, the DSSCs fabricated with a Ni_3_N CE showed a notable power energy conversion efficiency of 2.88% and remarkable stability. The prominent performance of the Ni_3_N thin film was ascribed mainly due to good conductivity, high electrochemically active sites with excellent 3D nano rhombus structures and high electrocatalytic activity. Overall, these results demonstrate that the Ni_3_N electrode is capable of being considered for efficient SCs and DSSCs. This investigation also offers an essential directive for the advancement of energy storage and conversion devices.

## Abstract 2

Self-supported 3D nano-rhombus (nano-diamond) shaped Ni_3_N coated on FTO glass which serves as a CE in DSSCs and supercapacitors. .

## Graphical abstract

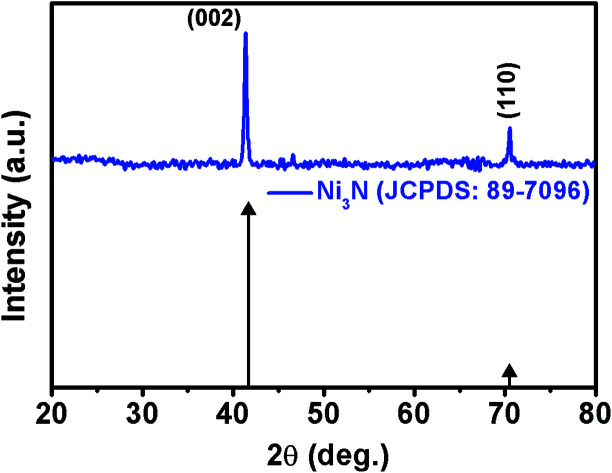

## 1. Introduction

The primary source of energy for our planet is solar radiation. The fossil fuels that are available today were created by photosynthesis of micro-plankton and other organisms millions of years ago. In addition, wind energy is created by the unequal heating of our planet by the sun. Conventional energy sources such as coal, fossil fuels and nuclear power are creating an energy crisis with environmental problems. Even hydropower dams are not free from green house emission.^1^ Increasing energy and environmental problems have urged researchers to focus on renewable energy resources. Solar energy is recognized as the most abundant energy source, which can rectify environmental and energy associated issues. Of the many types of solar cells, DSSCs are the cheapest, when compared to conventional silicon solar cells. However, DSSCs have short-comings such as low PCE and stability. Moreover, the usage of platinum (Pt) and other noble metal counter electrodes (CE) account for a significant portion of DSSC manufacturing costs.

Design and development of alternative CEs from different materials have attracted immense research interest in recent years. Many types of CEs have been investigated as alternatives for Pt CEs, such as transition metals (oxides, sulfides, and nitrides),^2–5^ carbon based materials (nanotubes,^6^ graphene oxide,^7^ C_60_, C_71_, and PCBM),^8,9^ and conjugated-polymers.^10–12^ The CE material must possess a superior conductivity to enable rapid transportation of electrons and robust electrocatalytic activity to reduce triiodide ions, which should be comparable if not surpass Pt CEs. Several metal oxides have been examined for photo and electrocatalytic activity, some of which are outlined below, and a one-step synthesis of a BiFeWO_6_/BiOI nanocomposite was investigated for photochemical activity by Malathi *et al.*^13^ Highly porous cobalt hydroxide (meso-Co-OH) was found to be a cost-effective CE for efficient oxygen evolution reactions with the advantage that the material can be synthesized using a simple precipitation method.^14^ Recently, transition metal sulfides and graphitic carbon nitrides have been extensively explored as catalytic materials for photoelectrochemical applications.^15–17^

In particular, transition metal nitride (TMN) based materials have high electrocatalytic activity which arises from a precious metal-like modified electronic cloud structure, due to this it has found a vast number of applications such as in (a) lithium ion batteries,^18^ (b) electrocatalytic applications^19,20^ (like hydrogen production,^21^ hydrogen and oxygen evolution reactions,^22,23^ solar cells, supercapacitors and many more) and (c) magnetic particles.^24^ TMNs have attracted a lot of research, and a few of them are listed below. MoN atomic size thin nanosheets were fabricated by Xia *et al.*^25^ TiN showed inherently better electrocatalytic activity. Many other nitride CEs have shown better activity than their oxide or pure form CEs.^6^ A simple two step, solid state reaction was used to synthesise cobalt molybdenum nitride (Co_0.6_M_1.4_N_2_) which is able to show excellent hydrogen evolution reaction (HER) activity in acidic conditions.^26^ Nickel is one of Earth’s abundant materials, and nickel based CE systems have been studied previously by various groups (NiO, NiS *etc.*),^27,28^ with excellent HER activity of NiMoN_*x*_/C being reported by Chen *et al.*^29^

Previously, Ni_3_N with a sponge-like structure was reported for electrocatalytic activity, but in this work the Ni_3_N was supported by being embedded in carbon–nitrogen material, which acts as a glue as well an electronic activator.^30^ The electrocatalytic activity of self-supported Ni_3_N was investigated *via* three electrode alkaline hydrogen evolution. Unlike previous studies, in this work the Ni_3_N was self-standing without the need for external support.^31^ Oxygen evolution reactions (OERs) were performed with a CE made of three nanolayers with carbon cloth at the bottom, above which a Ni_3_N nanoarray was grown to support a 3D Ni carbonate layer. Due to this architecture superior electrocatalytic activity was shown, even with very small over drive voltages (400 mV for 20 mA cm^−2^ in 1.0 M KHCO_3_ (bulk pH: 8.3)).^32^ Very little work has been reported on DSSC Ni_3_N CEs. In 2011, Park *et al.* used RF magnetron sputtering to deposit Ni–N films which were used as CEs for DSSCs (N719 dye as sensitizer and I_3_^−^/I^−^ as redox couple).^33^ A surface nitrided layer was engaged as the CE in DSSCs by Q. W. Jiang *et al.*^27^

Ni_3_N can also be utilized as an electrode in supercapacitors. Here are a few studies that have used Ni–N based materials for supercapacitor applications. Tiny Ni_3_N nanoparticles firmly fastened to graphene sheets were used as electrodes in supercapacitors, the results showed that the specific capacitance could get as high as 2087.5 F g^−1^ (at 1 A g^−1^), with a superior energy density of 50.5 W h kg^−1^ at 800 W kg^−1^.^34^ A 3D nickel nitride (Ni_3_N) nanosheet, consisting of a nanoflake structure was fabricated on carbon cloth *via* a hydrothermal process and had potential applications in the field of supercapacitors and flexible Li-ion batteries.^35^ It was found that high areal capacitance (around 655.1 mF cm^−2^) could be achieved from Cu_2_NiSnS_4_ nanoparticles embedded onto reduced 2D graphene.^36^

The development of Ni_3_N based nanomaterial technology plays an important role in energy harvesting and storage, because of the properties and features of the materials used. A single-electrode system for both SCs and DSSCs would be beneficial for smart energy storage and conversion devices. According to knowledge on the similarity of the electronic structures of TMNs and noble metals, it can be expected that Ni_3_N nitrides are capable of delivering Pt-like electrocatalytic performances. Consequently, an examination into the electrochemical behaviour of Ni_3_N is very important not only for exciting scientific interest, but also for securing low-cost substitute electrode materials for supercapacitors as well as for Pt in DSSCs.

In this study we present self-supported 3D nano-rhombus (nano-diamond) shaped Ni_3_N coated on FTO glass which serves as a CE in DSSCs and supercapacitors. To our knowledge this report is the first on Ni_3_N 3D nano-rhombus structured films for solar cell and supercapacitor applications.

## 2. Experimental section

### 2.1 Fabrication of nickel nitride (Ni_3_N) thin films

The nickel nitride (Ni_3_N) thin films were fabricated with a single step reactive RF magnetron sputtering technique. To fabricate this Ni_3_N thin film, a metallic nickel target (Ni) (Testbourne Ltd., UK, 2 inch dia., and 3 mm thickness) was used. Prior to the deposition, the substrates were cleaned ultra-sonically with water and acetone for 15 mins to remove the surface impurities. After the cleaning process, the substrates were loaded into the sputtering chamber and evacuated at the base pressure of 5 × 10^−3^ mbar. During Ni_3_N film deposition, the substrate temperature was kept at 200 °C with the argon (99.99%) and nitrogen (99.99%) gases held at 16 and 4 sccm respectively and an RF power of 80 watts applied to the Ni target. During deposition the distance between the target and the substrate was maintained at 60 mm. The Ni_3_N deposition duration was 60 min and the thickness of the Ni_3_N deposited films was about 400 nm.

### 2.2 Characterization of the films

The structural and phase formation of the as-deposited Ni_3_N thin film was observed *via* X-ray diffractometer (XRD-Bruker D8 Advance) measurements with Cu-Kα radiation (*λ* = 0.154 nm). The surface morphology of the Ni_3_N thin film was inspected using FESEM (FESEM-Carl Zeiss, Supra 55) and the elemental composition was analysed by EDS equipped with FESEM. The surface roughness and topography of the Ni_3_N thin film were examined using AFM (Bruker Dimension ICON).

### 2.3 Fabrication of DSSCs

The commercially available TiO_2_ coated FTO glass plates were treated with 40 mM TiCl_4_ solution for 30 min at 70 °C, and then subjected to a sintering process at 500 °C for 30 min. After cooling to 100 °C, the resultant substrates were dipped in dye solution under dark conditions for a dipping time of 16 h. N719 dye (Solaronix) was the dye used here, in a combination of *tert*-butanol/acetonitrile (1/1 v/v) at a concentration of 0.3 mM. The electrodes were then taken off and rinsed well with the appropriate solvents to remove the unanchored dye molecules from the surface of the TiO_2_ film, followed by drying under a N_2_ purge; the dye anchored active electrode area was 0.74 cm^2^. The DSSC test cells were assembled by similar methods as described in our recent report.^37^ The Ni_3_N based tin films were formed through a RF magnetron sputtering deposition method and incorporated as CEs in DSSCs with a standard photoelectrode used for open cell configurations.

The electrolytes E1, E2 were injected through a pre-drilled hole in the CE and the holes sealed with Kapton adhesive tape. The details of E1 and E2 are given below.

E1: DMPII (0.5 M)–LiI (0.1 M) – I_2_ (0.05 M)–GuNC (0.1 M)–4-*t*BP (0.5 M) in methoxypropionitrile (MPN).

E2: DMPII (0.5 M)–LiI (0.1 M) – I_2_ (0.05 M)–GuNC (0.1 M)–4-*t*BP (0.5 M) in acetonitrile (AcN).

The photocurrent density–voltage (*J*–*V*) measurement of the open structured test cells was conducted *via* a xenon arc solar simulator (PEC-L01, Peccell Inc., Japan) with an AM 1.5 spectral filter and a sourcemeter (2401N Keithley Instruments Inc.), its light intensity was maintained at 1 Sun condition (100 mW cm^−2^, AM 1.5 condition) using a calibrated mono-Sisolar cell. A mask of 0.25 cm^2^ (5 mm × 5 mm) was used for *J*–*V* measurements. Electrochemical impedance spectroscopy (EIS) measurement was performed *via* IVIUMSTAT in the frequency range of 0.1 Hz to 1 MHz.

## 3. Results and discussion

### 3.1 X-ray diffraction analysis

**Fig. 1:**
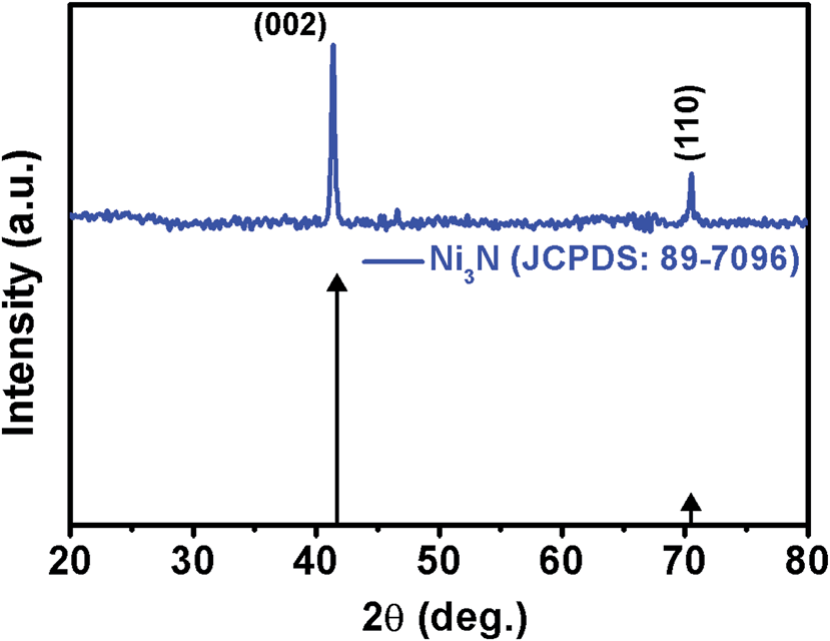
X-ray diffraction pattern of the as-deposited Ni_3_N thin film.

The purity and phase formation of the as-deposited Ni_3_N thin films were characterized using XRD spectroscopy and the obtained XRD pattern is displayed in Fig. 1. Diffraction peaks of Ni_3_N are observed at 2*θ* values of 41.60° and 70.37° which correspond to (002) and (110) diffraction planes of Ni_3_N. The exhibited diffraction peaks match a hexagonal Ni_3_N phase in concurrence with JCPDS card no. 89-7096. The average crystallite size, dislocation density and lattice strain were calculated according to previous reports.^38,39^ The crystallite size of Ni_3_N was calculated from (002) reflections and the obtained crystallite size of the Ni_3_N thin film is about 28.73 nm, the dislocation density (*δ*) is 1.2115 × 10^15^ lines per m^2^ and the calculated lattice strain (*ε*) is 0.003401.

### 3.2 Morphology and elemental analysis

**Fig. 2:**
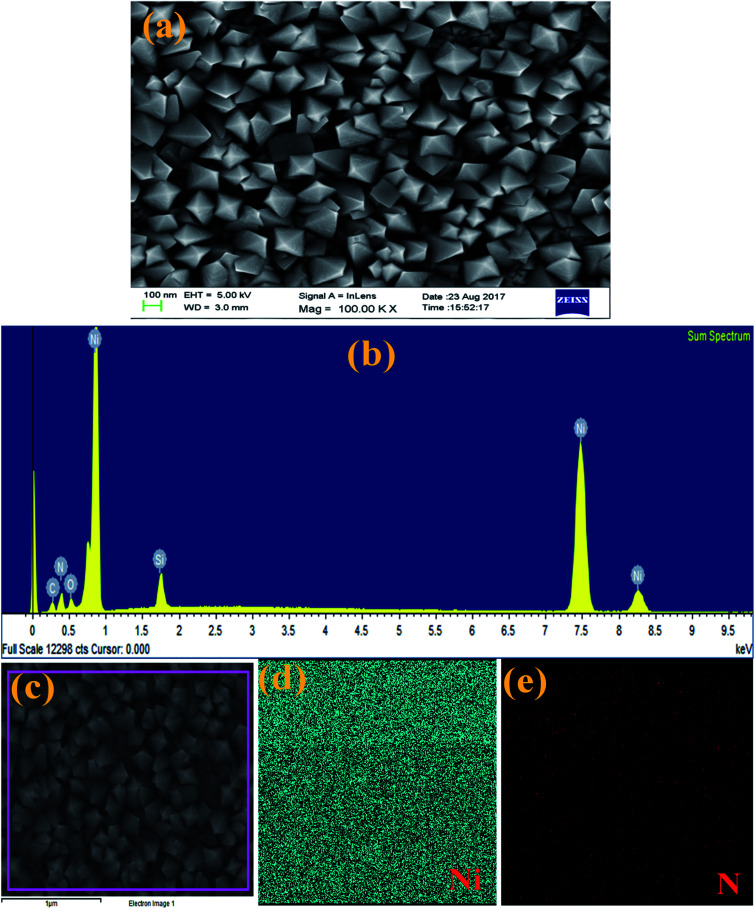
(a) FESEM image of Ni_3_N thin film, (b) EDS spectrum of Ni_3_N thin film, (c) secondary electron image of Ni_3_N thin film, and (d and e) EDS elemental mapping for Ni and N, respectively.

The surface morphology and elemental composition of the as-made Ni_3_N thin film were analysed using FESEM equipped with EDS. Fig. 2 represents the FESEM image and EDS spectrum of Ni_3_N thin film with consequent elemental mapping for Ni and N. The FESEM analysis clearly shows that the exhibited surface morphology of these Ni_3_N thin films is a 3D nano rhombus like structure (Fig. 2(a)). The displayed fine morphology mainly depends on fabrication route and reaction conditions. The well observed 3D nano rhombus like structure may be favourable for effective electron transport and advance the efficiency of supercapacitors and DSSCs.

The constituent elemental composition of Ni_3_N thin film was examined by EDS and the resultant spectrum is displayed in Fig. 2(b). These results confirm that the deposited film is composed of Ni and N elements with no other impurity peaks observed. The elemental mapping (Fig. 2(d and e)) of the Ni_3_N thin film demonstrates that the atomic distribution of Ni and N is uniform and further proves the presence of Ni and N atoms in the Ni_3_N thin film.

### 3.3 Surface topography analysis

**Fig. 3:**
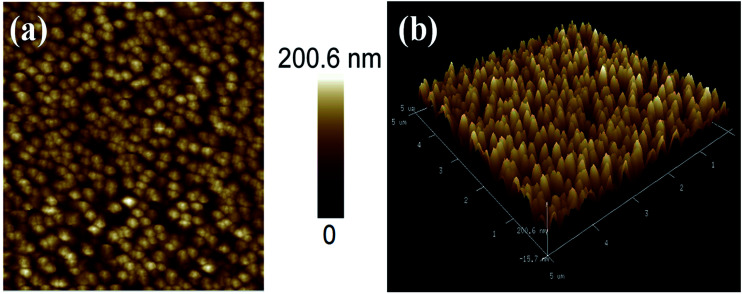
(a and b) 2D and 3D AFM micrographs of Ni_3_N thin film sputtered on Si substrate.

Atomic force microscopy (AFM) is an important tool for examining surface topography and roughness parameters of the thin films. The AFM micrographs of Ni_3_N films deposited on Si (100) substrates are shown in Fig. 3(a and b). The surface topography of the Ni_3_N film shows that grains are uniformly distributed, without any fractures or voids in the film’s surface. The surface topography provided by the AFM surface images is similar on comparison to FESEM results (FESEM image, Fig. 2(a)). The thin film surface roughness was also derived from AFM investigations. The root mean square roughness (*R*_q_) and the average roughness (*R*_a_) were found to be 68.4 nm and 56.2 nm, respectively.

### 3.4 Electrochemical measurements of the Ni_3_N electrode

**Fig. 4:**
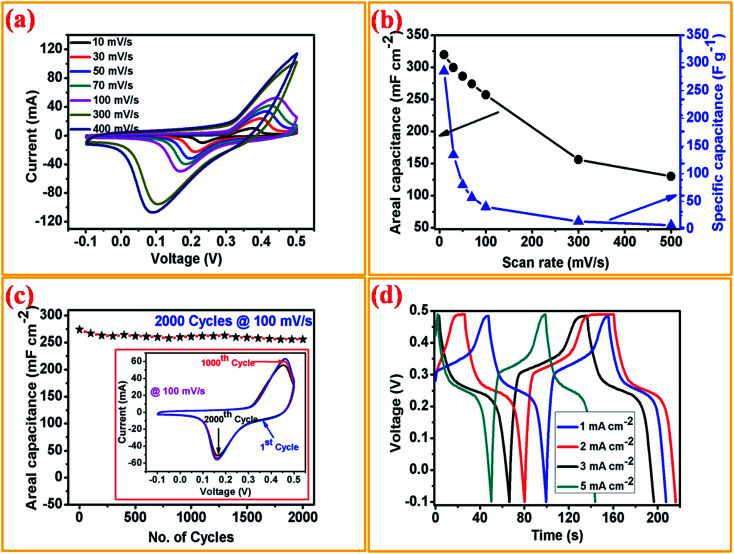
Electrochemical measurements of the Ni_3_N thin film electrode. (a) CV curves, (b) calculated areal capacitance and specific capacitance with respect to different scan rates, (c) the cycle performance of the Ni_3_N electrode carried out at the scan rate of 100 mV s^−1^ with calculated areal capacitance up to 2000 cycles (the inset figure represents the CV curves of the 1^st^, 1000^th^ and 2000^th^ cycles) and (d) charge–discharge profiles taken at different current densities.

In order to investigate the electrochemical supercapacitive performance of the as-prepared Ni_3_N thin film electrodes, CV and GCD measurements were performed. Fig. 4(a) depicts the CV curves of Ni_3_N using a three electrode cell configuration with 1 M KOH electrolyte. All of the CV curves were logged with a fixed potential of −0.1 to 0.5 V at a different scan rate ranging from 10 to 500 mV s^−1^. From the CV curves we observe two strong redox (anodic and cathodic) peaks, the anodic peaks are between 0.37 and 0.45 V with a similar trend for the cathodic peaks ranging from 0.08 to 0.2 V, as displayed in Fig. 4(a), which is due to the reversible redox reactions and good rate capabilities of the Ni_3_N electrode which is attributed to the capacitive characteristics produced by faradaic reactions. Hence the observed peak shifts mainly occur at a lower resistance, showing the pseudo-capacitive characteristic of the Ni_3_N electrode.^40,41^ All of the CV curves show a similar response even when the scan rate is increased to 500 mV s^−1^, this could be caused by fast ion and charge mobility in the KOH electrolyte.

The areal capacitance of the Ni_3_N electrode was derived from the integral area under the curve of the obtained CV curves using the following equation (eqn (1));^41^1
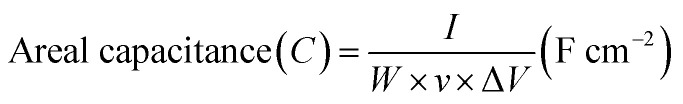
where, *I* represents the current in mA, *v* is the scan rate in mV s^−1^, *W* is the geometric area of the working electrode (1 × 1 cm^2^) and Δ*V* is the applied potential window in V. From CV analysis, the highest areal and specific capacitances are 319.5 mF cm^−2^ and 285 F g^−1^ at a scan rate of 10 mV s^−1^. After increasing the scan rate to 500 mV s^−1^, areal and specific capacitances of 130 mF cm^−2^ and 6.8 F g^−1^ are obtained. From CV analysis, as the scan rate increases from 10 to 500 mV s^−1^ the areal capacitance and specific capacitance gradually decrease, as shown in Fig. 4(b), this is because of the diffusion limit of the active electrolyte ions within the active surface of the electrode and, at high scan rates, the interfacial reaction kinetics.^41^ The calculated areal and specific capacitances with respect to the different scan rates is shown in Fig. 4(b). The cycling stability of the Ni_3_N electrode was investigated by CV analysis at a fixed scan rate of 100 mV s^−1^ up to 2000 consecutive cycles, with the corresponding capacitance retention plot shown in Fig. 4(c), a capacitance retention of 93.7% was obtained after 2000 CV cycles, suggesting that the as-prepared Ni_3_N electrodes show excellent electrochemical stability. To further understand the supercapacitive performance of the Ni_3_N electrode, galvanostatic charge–discharge (GCD) measurements were conducted at different current densities (1, 2, 3 and 5 mA cm^−2^). Fig. 4(d) shows the GCD curves of the Ni_3_N electrode with respect to the different constant current densities. In these GCD profiles a slight curvature is observed, hence this result indicates the influence of both redox reactions (pseudocapacitor) and electrical double layer (EDLC) response which is similar to trends previously reported.^40,42–44^

### 3.5 Electrocatalytic activity of Ni_3_N for triiodide reduction

**Fig. 5:**
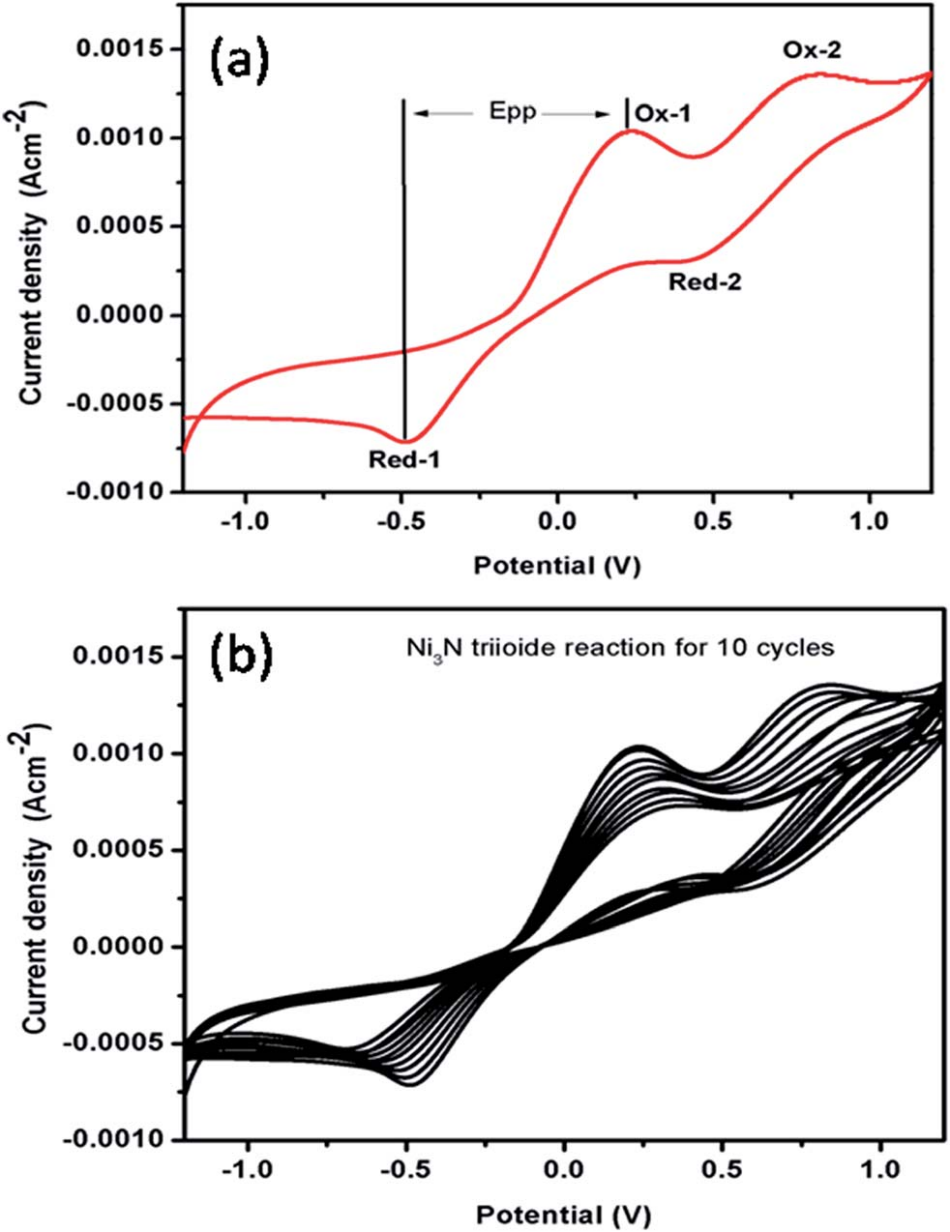
(a) Cyclic voltammogram of the Ni_3_N CE, and (b) stability test of the Ni_3_N CE at 10 cycles at a scan rate of 0.1 V s^−1^ in 10 mM LiI, 1 mM I_2_ and 0.1 M LiClO_4_ as the supporting electrolyte in acetonitrile.

The electrocatalytic performance of Ni_3_N CEs for the reduction of I_3_^−^ to I^−^ ions was observed by CV studies using three electrode configurations at a scan rate of 0.1 V s^−1^, with the resulting CV curve for the I^−^/I_3_^−^ redox species depicted in Fig. 5(a). It should be noted that there are two pairs of redox peaks (Ox-1/Red-1, Ox-2/Red-2) for the Ni_3_N electrodes. The left side pair (low potential region) of the redox peaks can be ascribed to the redox reaction shown in eqn (2), and the right side pair (high potential region) of the redox peaks can be associated with the redox reaction shown in eqn (3).^45,46^

2 I_3_^−^ + 2e^−^ ↔ 3I^−^

3 3I_2_ + 2e^−^ ↔ 2I_3_^−^

The Ni_3_N electrode CV curve profile is similar in shape to that of a conventional platinum electrode reported in a previous report,^45^ which indicates a similar electrocatalytic activity for Ni_3_N in a I^−^/I_3_^−^ redox system. The redox peaks, Ox-1 and Red-1, are the focus of our investigation in light of the fact that the CE is accountable for the electrocatalytic I_3_^−^ reduction in DSSCs.^47,48^ The peak current density and the peak-to-peak separation (*E*_PP_) are two vital factors for assessing the electrocatalytic performances of a CE. A higher *I*_PC_ implies a quicker reaction rate while a lower *E*_PP_ implies a smaller over-potential and enhanced electrocatalytic behaviour for I^−^/I_3_^−^ redox reactions. Hence, a higher *I*_PC_ and a lower *E*_PP_ are advantageous for a Ni_3_N CE to accomplish good electrocatalytic activity.

Furthermore, the electrocatalytic stability of a catalyst towards the reduction of I_3_^−^ to I^−^ ions is an important parameter for possible DSSC applications. To examine the electrocatalytic stability of the fabricated Ni_3_N electrode, the electrocatalytic test was repeated for I_3_^−^ reduction and the obtained results are depicted in Fig. 5(b). It should be noted that after 10 runs, the electrocatalytic activity of the Ni_3_N electrode was mostly maintained at that of its initial run, demonstrating a good electrocatalytic stability.

### 3.6 Photovoltaic performance of the DSSCs

**Fig. 6:**
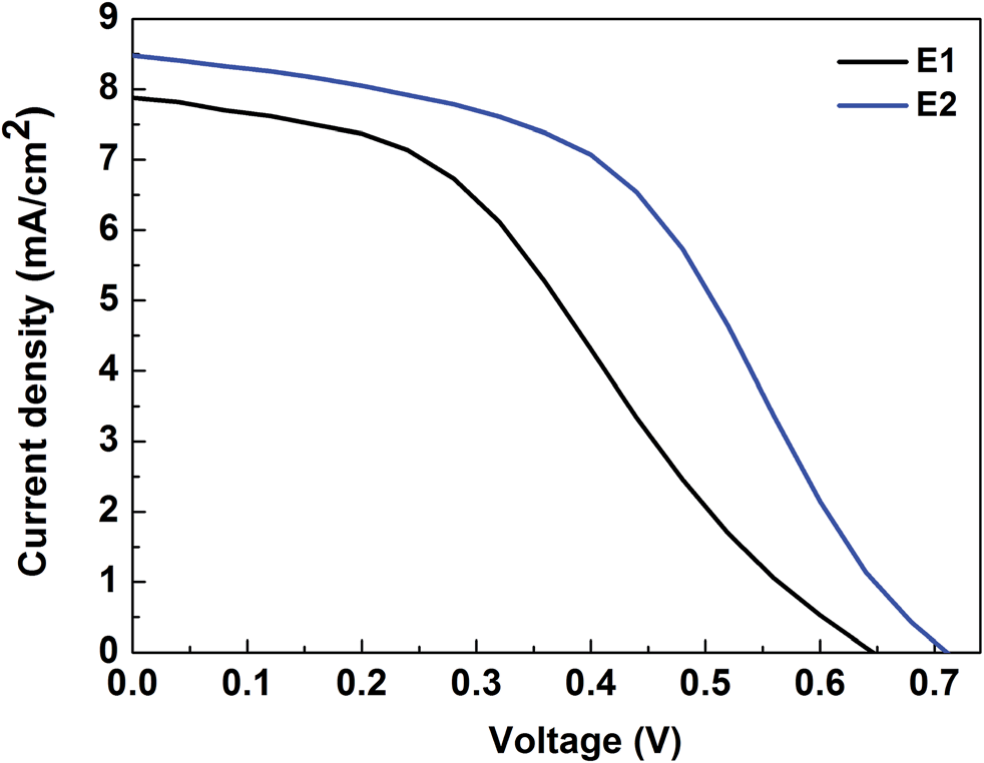
Photocurrent density–voltage (*J*–*V*) characteristic performance of open test devices.

Fig. 6 reveals the *J*–*V* characteristics of the DSSC with NiN CEs in E1 and E2 based ionic liquid electrolytes. The photovoltaic parameter performances are tabulated in Table 1. The DSSC with methoxypropionitrile (MPN) based electrolyte (E1) exhibits a lower conversion efficiency (*η*) of 1.96% compared to acetonitrile (AcN) electrolyte (E2) of *η* = 2.88%. This lower conversion performance is affected by poor fill factors and is strongly associated with the inferior electrocatalytic activity of Ni_3_N CEs in iodide redox electrolyte. The poor short-circuit current density and fill factor of Ni_3_N CEs are reflected in its performance. Nevertheless, the *J*_sc_ of the fabricated test device is higher in E1 electrolyte which is mainly due to the volatile AcN based solvent. Increased short-circuit current density (*J*_sc_) and fill factor are reflected in an increased conversion efficiency with E1 compared to E2 which may be due to higher migration and penetration of AcN electrolyte into porous TiO_2_ and the higher conductivity nature of the AcN solvent compared to MPN.

**Table 1:** Photovoltaic performance parameters of open test devices

Electrolytes	*V*_oc_ (V)	*J*_sc_ (mA cm^−2^)	FF	*η* (%)
E1	0.65	7.87	0.39	1.96
E2	0.71	8.47	0.47	2.88

**Fig. 7:**
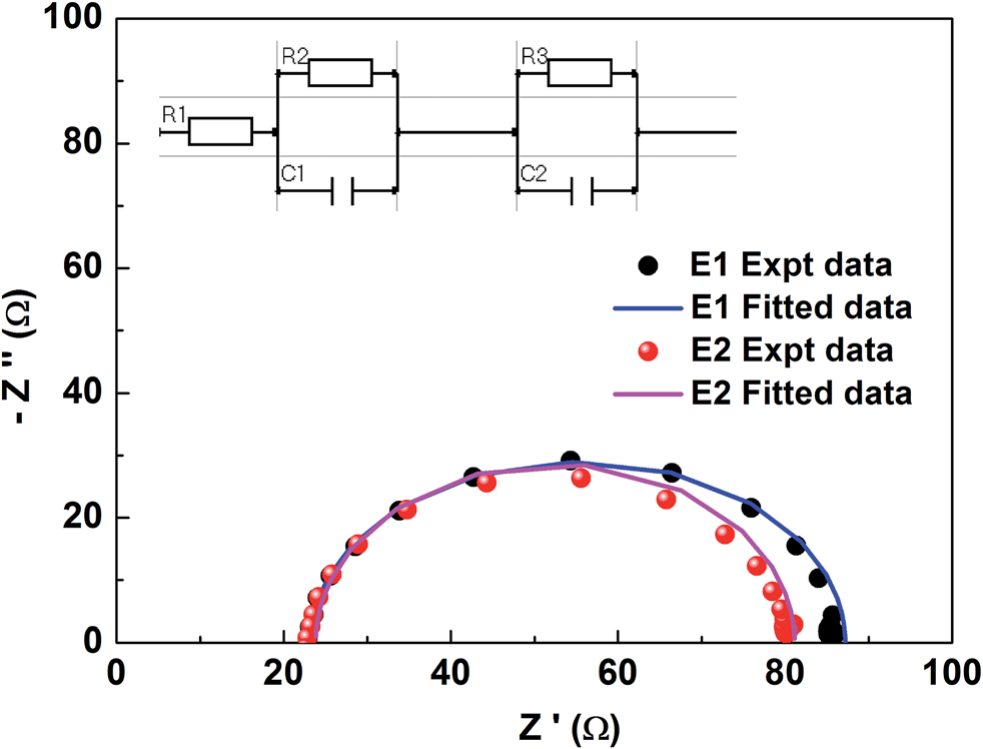
EIS study performance of dummy cells with E1, E2 electrolytes.

The test device performance accorded well with EIS studies. An impedance spectrum was recorded for the fabricated DSSC devices with Ni_3_N CEs in the two different electrolytes (E1 and E2). The EIS measurements were performed under light using a COMPACTSTAT. The Nyquist plots of the electrodes are presented in Fig. 7, and the equivalent circuit model is fitted, comprising *R*_s_ and *R*_CT_. The series resistance (*R*_s_) which, due to contact resistance of Ni_3_N with the FTO plate, and charge transfer resistances *R*_CT1_ and *R*_CT2_, signifies interfacial charge transfer resistances at the CE/electrolyte interface for an I^−^/I_3_^−^ redox reaction and the photoelectrode (PE)/electrolyte interface (*i.e.* impedance at the electrolyte/electrode interface). These resistances, which were obtained after fitting the impedance spectra well with IVIUMSOFT software, are tabulated in Table 2. The lower resistance values observed for E2 electrolyte are reflected in the device performances.

**Table 2:** EIS parameters of open dummy test cells in electrolytes E1 and E2

Parameter	E1	E2
*R*_s_ = *R*_1_ (Ω)	23.8	23.2
*R*_CT1_ = *R*_2_ (kΩ)	68.1	30.6
*R*_CT2_ = *R*_3_ (kΩ)	57.3	33.4
*C*_1_ (μF)	4.35	2.08
*C*_2_ (μF)	1.55	4.76

In the present work, the device performance of Ni_3_N CEs with an iodide based redox couple has been investigated. Further studies examining the conversion performance of these CEs with other redox couples may be worthwhile.

## 4. Conclusions

In conclusion, 3D nano rhombus Ni_3_N thin films were prepared using a reactive RF magnetron sputtering technique. The as-deposited Ni_3_N thin films were characterized by XRD, FE-SEM, AFM and EDS analysis. Subsequently, the as-deposited 3D nano rhombus Ni_3_N thin films were evaluated as active electrode materials in SCs and as a platinum free, low cost CE in DSSCs. The electrochemical SC behaviour of the Ni_3_N electrode was examined by CV and GCD. It demonstrated an areal capacitance value of 319.5 mF cm^−2^ at a lower scan rate of 10 mV s^−1^. Interestingly; the fabricated DSSCs with Ni_3_N CEs achieved a power energy conversion efficiency of 2.88% with a *V*_oc_ of 0.71 V, *J*_sc_ of 8.47 mA cm^−2^ and FF of 0.47. As a result, this 3D nano rhombus Ni_3_N showed an enhanced specific capacitance with excellent stability and high electrocatalytic activity as an efficient CE for DSSCs. These results suggest a potential application of 3D nano rhombus Ni_3_N thin films in electrochemical energy storage and conversion devices.

## Conflicts of interest

There are no conflicts to declare.

## Supplementary Material
